# Idiopathic Scrotal Calcinosis in Young Adults: Two Case Reports

**DOI:** 10.7759/cureus.109499

**Published:** 2026-05-23

**Authors:** Soufiane Laknizi, Mounir Jamali, Bouzid Balla

**Affiliations:** 1 Urology, Military Hospital Moulay El Hassan, Guelmim, MAR

**Keywords:** dermal calcification, histopathology, idiopathic scrotal calcinosis, scrotal nodules, surgical management

## Abstract

Idiopathic scrotal calcinosis (ISC) is a rare benign condition characterized by the gradual development of multiple painless calcified nodules confined to the scrotal skin. Although its exact origin remains poorly understood, several pathogenic mechanisms have been proposed, and its etiology continues to be debated. We report two cases of ISC in young adult males presenting with slowly progressive scrotal nodules, highlighting the characteristic clinical features of this uncommon entity, the role of surgical excision as definitive treatment, and the importance of histopathological examination in establishing the diagnosis.

## Introduction

Scrotal calcinosis is a rare benign disorder of calcinosis cutis characterized by the progressive development of firm, painless calcified papules or nodules confined to the scrotal skin. Its exact incidence and prevalence remain poorly defined, as the literature mainly consists of case reports and small series. The condition is usually described in young to middle-aged men, although cases in older patients have also been reported. Clinically, the lesions enlarge slowly over several years and may occasionally be associated with discomfort, pruritus, ulceration, or chalky discharge [1].

Although surgical excision is considered the treatment of choice and allows definitive histopathological confirmation, the pathogenesis remains controversial. Proposed mechanisms include primary idiopathic calcium deposition, dystrophic calcification of pre-existing epidermoid cysts, eccrine epithelial cyst or duct degeneration, and dartos muscle degeneration [2]. Differential diagnoses include epidermal inclusion cysts, steatocystoma, lipoma, angiokeratoma, solitary neurofibroma, genital leiomyoma, and scleroderma-related calcinosis [3]. We report two cases of idiopathic scrotal calcinosis in young adult males to highlight the clinical and histopathological features of this uncommon entity and to contribute to the ongoing debate regarding its origin.

## Case presentation

Case number one

**Figure 1:**
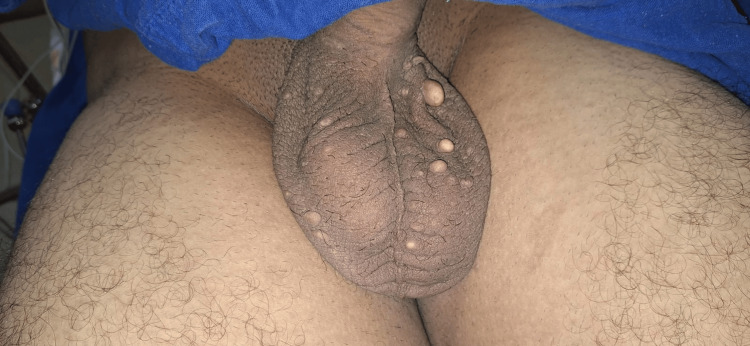
Preoperative appearance of the scrotum

A 32-year-old unmarried male with no significant past medical history presented to the Urology Department with a two-year history of painless scrotal nodules. He had no history of scrotal trauma, local infection, dermatological disease, previous surgery, or similar familial condition. These lesions had progressively increased in size. Physical examination revealed multiple firm, pearly-white scrotal nodules, the largest measuring 1.5 cm (Figure 1). The nodules were hard and mobile relative to the deep tissue planes. The remainder of the physical examination was unremarkable.

**Figure 2:**
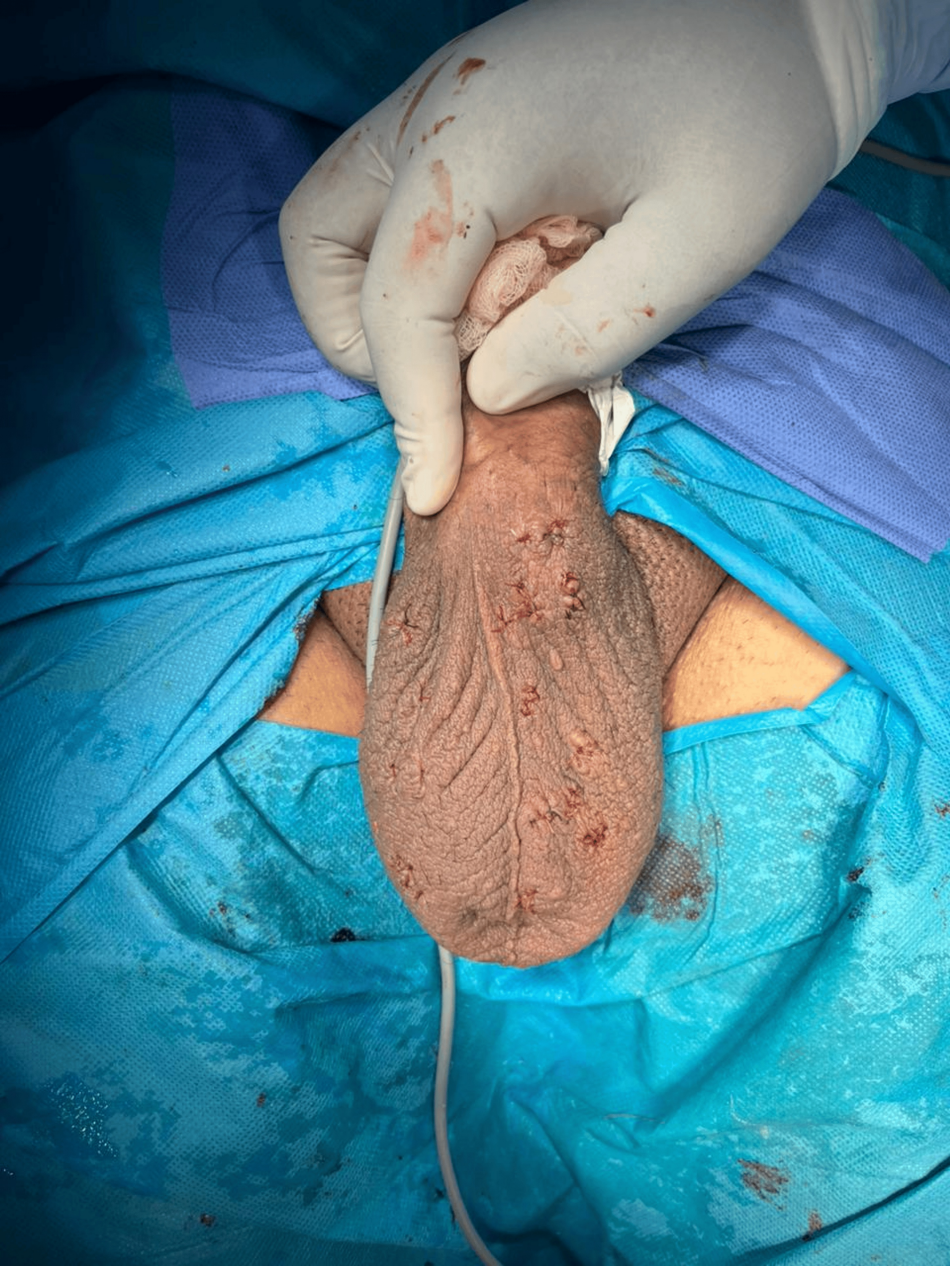
Immediate postoperative appearance following surgical excision

**Figure 3:**
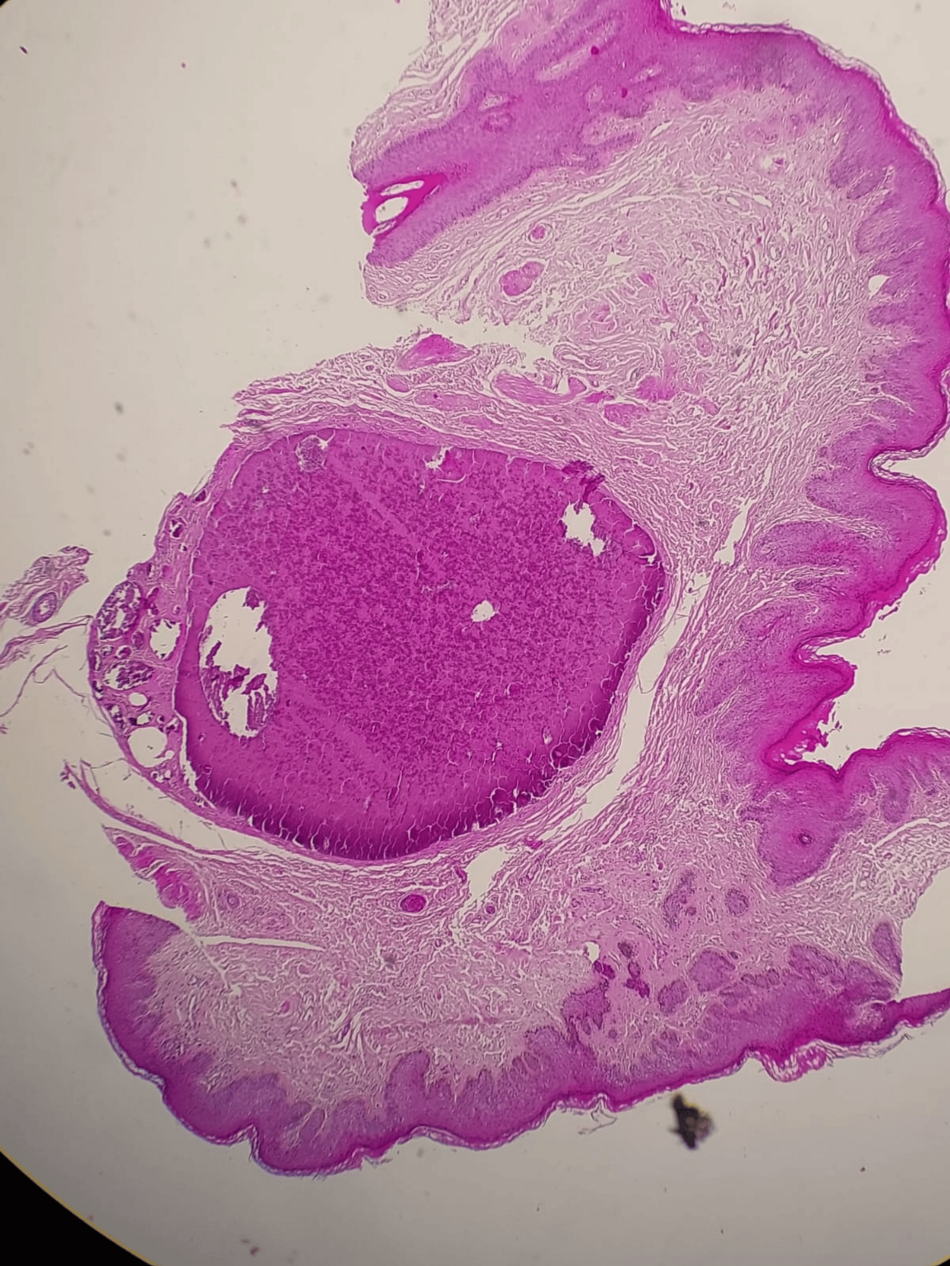
Calcified intradermal nodule devoid of epithelial lining (H&E, 40×)

Laboratory investigations, including serum calcium, phosphate, urea, and creatinine levels, were within normal limits (Table 1). The patient underwent complete surgical excision of the scrotal lesions under spinal anesthesia (Figure 2). Histopathological analysis of the specimen revealed several nodular foci of calcification containing amorphous material. These were bordered in areas by a foreign-body giant-cell resorptive infiltrate, with no evidence of malignancy (Figure 3). These findings confirmed the diagnosis of idiopathic scrotal calcinosis. The postoperative course was uneventful. At the one-month follow-up, wound healing was satisfactory, the cosmetic outcome was considered good, and no recurrence was observed.

**Table 1:** Laboratory findings in both cases, showing normal phosphocalcic and renal parameters

Laboratory Investigation	Case 1	Case 2	Normal Range
Serum Parathyroid Hormone (PTH)	5.9 pmol/L	5.5 pmol/L	1.6–6.9 pmol/L
Serum Calcium	2.47 mmol/L	2.27 mmol/L	2.10–2.54 mmol/L
Serum Phosphate	1.19 mmol/L	1.45 mmol/L	0.74–1.52 mmol/L
Blood Urea	4.3 mmol/L	3.5 mmol/L	3.0–9.2 mmol/L
Serum Creatinine	80 µmol/L	72 µmol/L	64–111 µmol/L

Case number two

**Figure 4:**
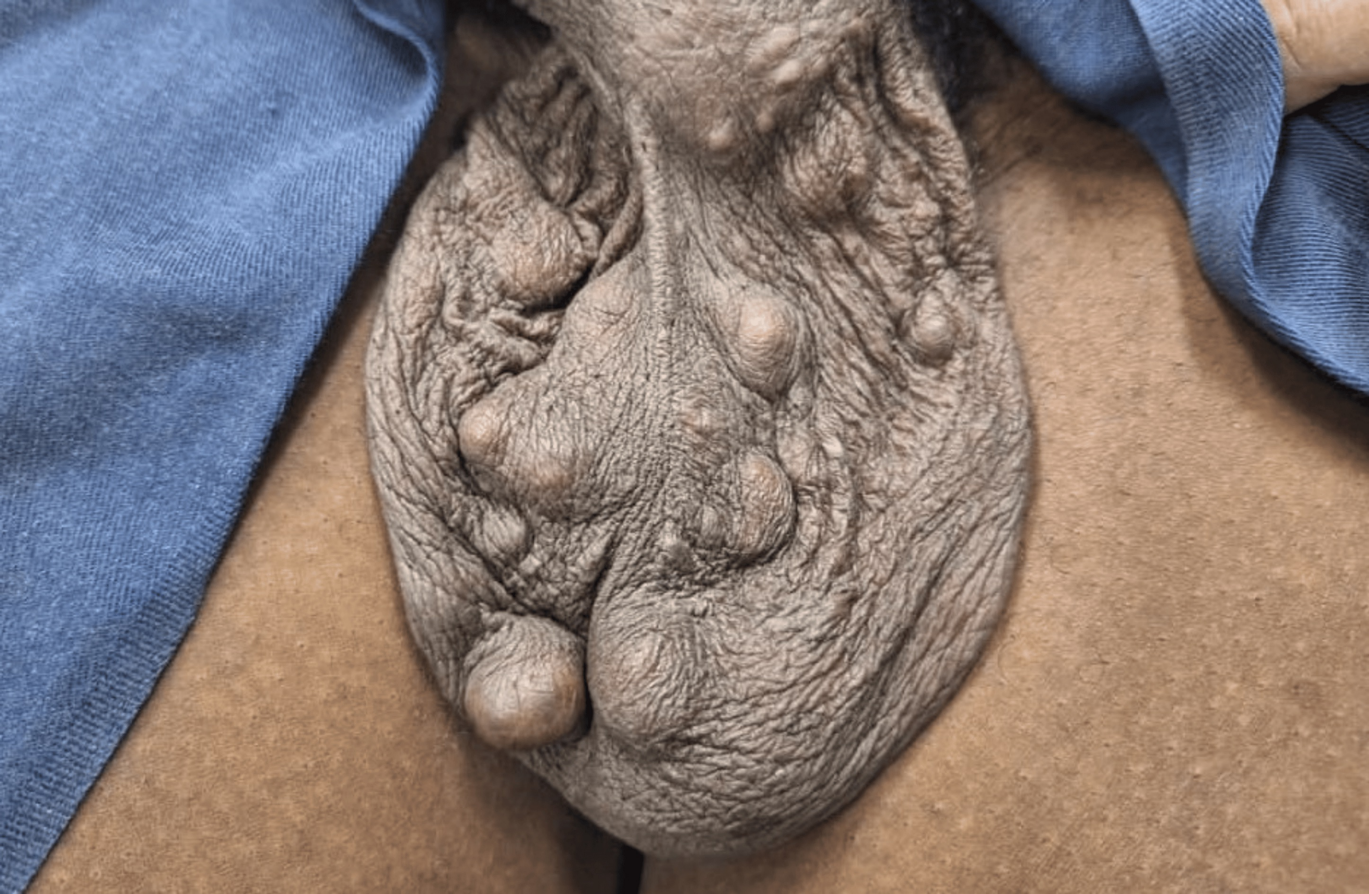
Preoperative appearance of the scrotum in case number two

A 33-year-old unmarried male with no relevant medical history presented with bilateral, painless scrotal enlargement evolving over four years. He reported no history of scrotal trauma, local infection, dermatological disorder, previous surgical intervention, or familial occurrence of similar lesions. The scrotum was studded with nodules ranging from 0.5 cm to 2 cm in diameter (Figure 4). These were firm, mobile, and exhibited a whitish central appearance. No other cutaneous lesions were identified elsewhere on the body. Serum calcium and phosphate levels were normal (Table 1).

**Figure 5:**
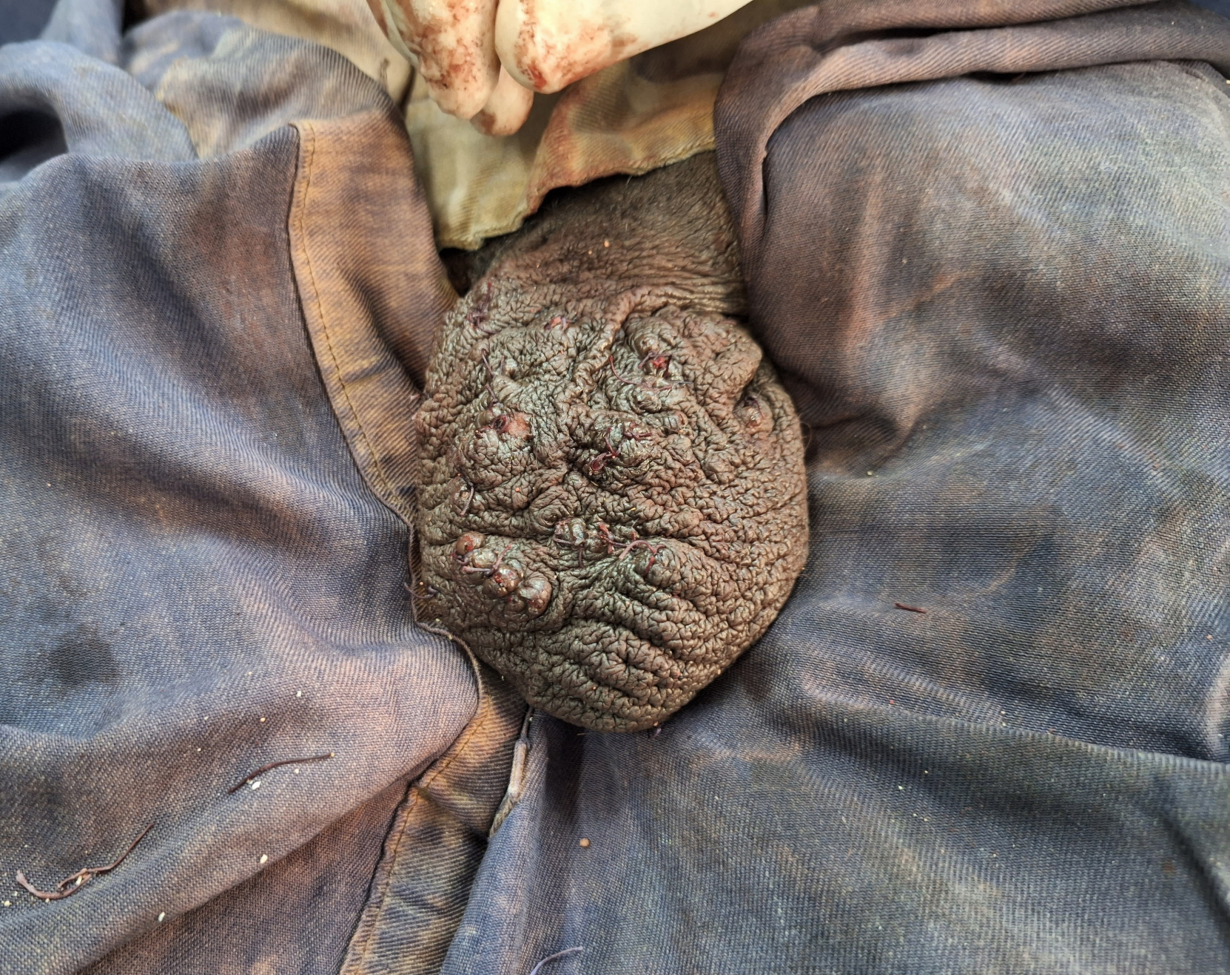
Immediate postoperative appearance in case number two

**Figure 6:**
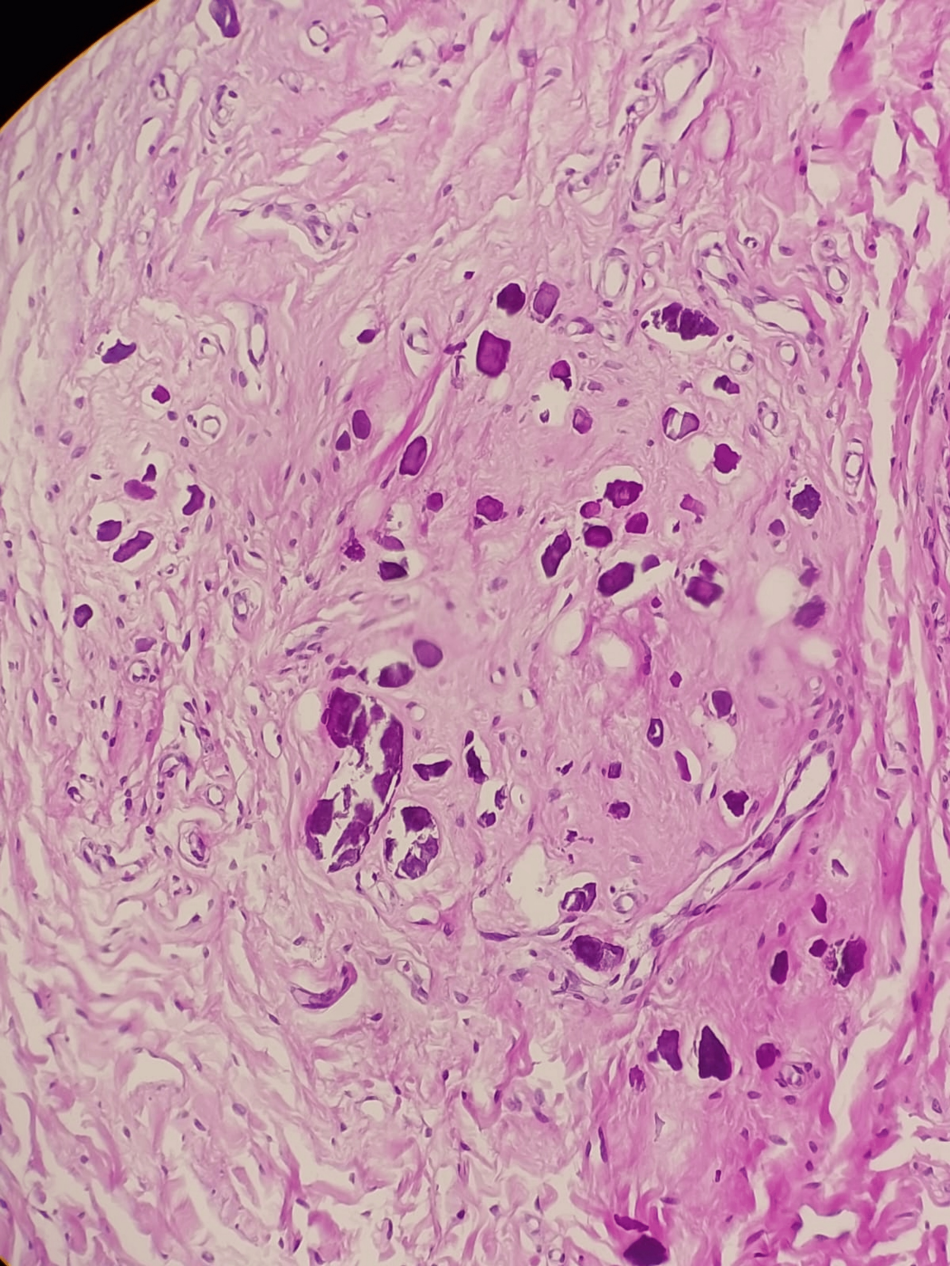
Granular as well as large globular basophilic deposits of calcium within the dermis (H&E, 200×)

Under local anesthesia, surgical removal of the scrotal nodules was performed (Figure 5). Histopathological examination showed variably sized dermal cystic spaces containing granular and large globular basophilic calcium deposits. A surrounding foreign-body granulomatous reaction composed of stromal cells, chronic inflammatory infiltrates, scattered histiocytes, and multinucleated giant cells was noted, with focal areas of irregular squamous epithelial lining. These findings were consistent with scrotal calcinosis (Figure 6). One month after surgery, the patient showed complete wound healing with a satisfactory aesthetic result. No evidence of recurrence was noted during this follow-up period.

## Discussion

Idiopathic scrotal calcinosis is an exceedingly rare and benign entity. It manifests as solitary or multiple calcified nodules, which are typically asymptomatic and localized strictly to the scrotum. While it generally occurs during the third decade of life, patients often seek medical consultation years or even decades after the initial onset, usually in the absence of any systemic disorders of calcium or phosphorus metabolism [3]. Although it is the most common form of genital calcinosis, similar lesions have been documented in females as vulvar calcinosis [4].

The main scientific issue in scrotal calcinosis remains its pathogenesis. Although the term “idiopathic” has traditionally been used when no definite cause is identified, this designation remains debated. Swinehart et al. suggested that scrotal calcinosis may represent the end stage of dystrophic calcification of epidermoid cysts [5]. Saad et al. also argued that epidermal inclusion cysts may represent the underlying abnormality rather than a purely idiopathic process [6]. Similarly, Parlakgumus et al. reported scrotal calcinosis related to resorption of cyst walls, supporting the concept that a cystic structure may disappear over time, leaving only dermal calcium deposits [7]. More recently, Li et al. reported a clinicopathological series of 14 cases and concluded that at least some cases may originate from epidermoid cysts [8].

Other pathogenetic mechanisms have also been proposed. Ito et al. described dystrophic scrotal calcinosis originating from benign eccrine epithelial cysts, suggesting that eccrine ductal structures may be involved in some cases [9]. Pabuççuoğlu et al. proposed another mechanism based on degeneration and necrosis of the dartos muscle followed by dystrophic calcification [10]. A possible role of nanobacteria in extraskeletal calcification has also been mentioned, but this hypothesis remains speculative and was not investigated in our patients [11]. Taken together, these theories suggest that scrotal calcinosis may not represent a single uniform disease, but rather a final common histological pattern of dermal calcium deposition resulting from different local processes.

Clinically, scrotal calcinosis may mimic several benign scrotal or cutaneous lesions. The differential diagnosis includes epidermoid cysts, steatocystoma, calcified sebaceous cysts, pilomatricoma, calcified onchocercoma, solitary neurofibroma, ancient schwannoma, lipoma, fibroma, angiokeratoma, lymphangioma circumscriptum, and, more rarely, scrotal neoplasms [3]. Histopathological examination is therefore essential, not only to confirm the diagnosis and exclude malignancy, but also to look carefully for epithelial remnants, cyst wall structures, keratin material, or ductal differentiation, which may help clarify the underlying pathogenesis [4].

Complete surgical excision remains the treatment of choice. It allows removal of the lesions, improvement of local discomfort or cosmetic concern, and definitive histopathological confirmation [11]. The surgical approach should be adapted to the number, size, and distribution of the nodules. In limited forms, local excision with preservation of uninvolved scrotal skin is usually sufficient. In extensive or massive forms, wider excision, staged procedures, subtotal excision of the involved scrotal wall, scrotoplasty, or other reconstructive approaches may be required to preserve scrotal function and obtain an acceptable cosmetic result [12,13].

Postoperative recurrence remains difficult to quantify because most published data consist of case reports or small series with variable follow-up. Recurrence appears uncommon after complete excision, but delayed recurrence has been reported, particularly when small residual nodules remain. For this reason, complete removal of all clinically visible lesions and long-term follow-up are advisable [14].

## Conclusions

Although rare, scrotal calcinosis should be recognized as a distinctive benign entity presenting as slowly progressive, painless scrotal nodules. Prompt clinical recognition, followed by complete surgical excision and careful histopathological examination, remains fundamental to accurate diagnosis and appropriate management. In the present cases, the histological finding of squamous epithelial remnants in one patient suggests that some lesions may reflect late dystrophic calcification of pre-existing epidermoid cysts rather than a purely idiopathic process. Thorough excision of all clinically evident lesions is essential to reduce the risk of recurrence, while timely intervention generally provides satisfactory cosmetic outcomes and patient reassurance.
